# 1120. Descriptive Analysis of Monoclonal Antibody Administration for Pregnant Patients with SARS-CoV-2 Infection

**DOI:** 10.1093/ofid/ofac492.959

**Published:** 2022-12-15

**Authors:** Praveen Kumar Vikraman, Kai Ming Chang, Thien-Ly Doan, Henry Donaghy

**Affiliations:** Northwell Health/ Northshore University Hospital, Englewood, New Jersey; Northwell Health/ Northshore University Hospital, Englewood, New Jersey; Long Island Jewish Medical Center, New Hyde Park, New York; Northwell Health, New Hyde Park, New York

## Abstract

**Background:**

We studied the safety and efficacy of the use of monoclonal antibodies (MAB) against SARS-CoV-2 in pregnant women who developed COVID-19 infection.

**Methods:**

We conducted a cross-sectional descriptive multi-center study of pregnant patients who developed SARS-CoV-2 infection from January 2021 to January 2022 and received MAB therapy. Primary outcomes assessed were infusion-related adverse events and pregnancy outcomes within one month of MAB infusion. The secondary outcomes assessed were hospitalization and ICU admission for COVID19 infection and thirty-day all-cause mortality.

**Results:**

141 patients were included in the study (median age 33 +/- 5.3 SD, median BMI 28.9 +/- 8.42 SD). In terms of COVID vaccination status, 49.6% received one dose, 36.1% were fully vaccinated, and 7% received the booster dose. Most patients received casirivimab/imdevimab (105, 74.5%) followed by sotrovimab (33, 23.4%). Four patients developed adverse reactions to MAB infusion (two grade-2 reactions and two grade-1 reactions as per the National cancer institute infusion reaction grading criteria). Only one patient (0.7%) was hospitalized for COVID-19 infection, however, she was not hypoxic nor required ICU admission. Five patients delivered within four weeks of MAB administration, however, four of those patients were of gestational age > 37 weeks. Data for 30-day all-cause mortality was available on 88.7% (125) of the patients and data for 30-day pregnancy adverse outcomes was available on 86.5% (122) of the patients due to lack of follow-up within the Health System. There was no reported 30-Day all-cause mortality within the cohort. Two patients (1.4%) had premature rupture of the membrane and one patient (0.7%) had premature delivery within 30 days of receiving MAB. Two patients had preeclampsia (1.4%) and one patient (0.7%) was admitted for evaluations of decreased fetal movements.

**Conclusion:**

Administration of monoclonal antibodies against SARS-CoV-2 was well tolerated during pregnancy. Only 4 out of 141 (2.8%) had mild to moderate infusion-related reactions. The 30-day pregnancy adverse outcomes observed were well below the mean background rate. There was no reported mortality among MAB recipients and only one patient was hospitalized for mild COVID19 infection.

**Disclosures:**

**All Authors**: No reported disclosures.

